# Tumor load rather than contrast enhancement is associated with the visual function of children and adolescents with optic pathway glioma – a retrospective Magnetic Resonance Imaging study

**DOI:** 10.1007/s11060-021-03941-1

**Published:** 2022-01-07

**Authors:** Anna Kilian, Annette Aigner, Michèle Simon, Daniel J. Salchow, Cornelia Potratz, Ulrich-Wilhelm Thomale, Pablo Hernáiz Driever, Anna Tietze

**Affiliations:** 1 Charité - Universitätsmedizin Berlin, corporate member of Freie Universität Berlin and Humboldt-Universität zu Berlin, Institute of Neuroradiology, Augustenburger Platz 1, 13353 Berlin, Germany; 2 Charité - Universitätsmedizin Berlin, Institute of Biometry and Clinical Epidemiology, Berlin, Germany; 3 Charité - Universitätsmedizin Berlin, Department of Pediatric Oncology and Hematology, Berlin, Germany; 4 Charité - Universitätsmedizin Berlin, Department of Ophthalmology, Berlin, Germany; 5 Charité - Universitätsmedizin Berlin, Department of Pediatric Neurology, Berlin, Germany; 6 Charité - Universitätsmedizin Berlin, Department of Pediatric Neurosurgery, Berlin, Germany; 7 German HIT-LOGGIC-Registry for Children and Adolescents With Low-Grade Glioma, Berlin, Germany

**Keywords:** Optic pathway glioma, Neurofibromatosis type 1, Magnetic Resonance Imaging, Gadolinium-based contrast agents, Visual acuity

## Abstract

**Introduction:**

Optic pathway gliomas are often asymptomatic tumors occurring in children with neurofibromatosis type 1 (NF1 + OPG) or sporadically (spOPG). Treatment is usually prompted by visual loss and/or tumor progression on MRI. The aim of this study was to investigate the relationship between visual acuity (VA), tumor growth, and contrast enhancement to provide more distinct indications for the administration of gadolinium-based contrast agents.

**Methods:**

Tumor load was retrospectively measured and enhancement semi-quantitatively scored on 298 MRIs of 35 patients (63% NF1 + OPG). Spearman rank correlation between tumor load and enhancement was calculated and a linear mixed model used to examine the influence of tumor load and enhancement on corresponding VA tests (LogMAR).

**Results:**

The optic nerve width in NF1 + OPGs was strongly associated with VA (regression coefficient 0.75; confidence interval 0.61—0.88), but weakly with enhancement (0.06; −0.04—0.15). In spOPGs, tumor volume and optic nerve width were more relevant (0.31; −0.19—0.81 and 0.39; 0.05—0.73) than enhancement (0.09; −0.09—0.27).

**Conclusions:**

Tumor load measures may be more relevant for the surveillance of optic pathway gliomas than enhancement, given that VA is the relevant outcome parameter. Regular contrast administration should therefore be questioned in these patients.

**Supplementary Information:**

The online version contains supplementary material available at 10.1007/s11060-021-03941-1.

## Background

With a prevalence of 1:3,000, Neurofibromatosis type 1 (NF1) is one of the most common cancer disposition syndromes [1]. Most frequently, tumors arise in the anterior optic pathway at young age, i.e. usually in children under 6 years, causing visual impairment and other visual symptoms [2]. Histologically, these tumors represent pilocytic astrocytomas (WHO grade I) in the majority of NF1 patients and are termed optic pathway gliomas (NF1 + OPGs). Their clinical course is often indolent [3], which contrasts that of patients with sporadic optic pathway gliomas (OPGs) in NF1-negative patients (spOPG) whose visual function is generally more severely affected and who present more commonly with central nervous system (CNS) symptoms such as increased intracranial pressure and hydrocephalus [4].

Monitoring children with both NF1 + OPGs and spOPGs aims at detecting visual loss and potential tumor growth as early as possible in order to start treatment in time. Therefore, visual acuity (VA) testing and MRI are performed on a regular base. To date, the determining factors to initiate chemotherapy, surgical tumor reduction, or radiotherapy are not well defined. In order to understand risk factors requiring intervention, a cohort of 83 NF1 + OPG patients was reassessed retrospectively in a multidisciplinary international workshop only recently [5]. The main finding was that visual deterioration and tumor growth on imaging are usually therapy drivers, aiming to preserve or improve the visual function. MRI is regarded as one diagnostic cornerstone in OPG patients.

Given the assumption that contrast enhancement is associated with tumor activity, MRI is usually performed with and without gadolinium-based contrast agents (GBCAs) [6]. In our experience as well as according to the recent literature [5, 7, 8], contrast enhancement is rarely the decisive criterion for treatment initiation. Especially in light of the yet undetermined relevance of gadolinium retention in the CNS, bone, and skin after repeated GBCA administrations [9], GBCAs should be applied with caution. This is especially relevant for children, because they can accumulate gadolinium over a longer period, and repeated exposures in this age group may be more harmful, as the skeleton and organs are still under development. The preferred site for gadolinium deposits are the globus pallidus and the dentate nucleus, which may increase the risk for future movement disorders or cognitive problems. The degree of gadolinium retention depends on the frequency of exposures and the type of chelate used. Linear molecules release the toxic gadolinium more readily, whereas the retention appears to be lower in macrocyclic compounds. But ultimately, gadolinium depositions in particularly CNS tissue have been detected regardless the type of GBCA, the patients’ renal function, or the integrity of the blood–brain-barrier [10, 11]. In addition, the insertion of an intravenous line may be unnecessarily stressful for affected children who are already burdened with regular hospital visits. Finally, additional costs for the health care system caused by GBCAs must be justified.

Only recently, Azizi et al. identified VA deterioration and tumor growth as primary determinants to initiate treatment in NF1 + OPG patients [5]. Contrast enhancement as a variable influencing visual function or as a decisive factor for treatment initiation was not investigated [5]. The Response assessment in Pediatric Neuro-Oncology (RAPNO) working group recommends GBCA administration as an integral part of the MRI surveillance protocol in low-grade gliomas, although changes of contrast enhancement are not a criterion for response or progression [12]. While in some pediatric low-grade gliomas contrast enhancement may facilitate their delineation or dissemination, this does not necessarily apply for optic pathway gliomas. The objective of the present study was therefore to investigate the association between contrast enhancement, tumor volume, and visual function in NF1 + OPG and spOPG patients to refine indications for GBCA administration. We hypothesized that (i) intracranial tumor volume, optic nerve width, or VA are not associated with contrast enhancement and that (ii) the T2w tumor volume, optic nerve width, and extension rather than the degree of contrast enhancement is associated with the VA. Either hypotheses were considered for NF + OPGs and spOPGs separately.

## Material and methods

### Patients

In this retrospective analysis, all patients under the age of 18 years diagnosed with or treated for an OPG at Charité Universitätsmedizin Berlin between 2004 and 2020 were included. Inclusion criterion was the availability of MRI data containing contrast enhanced series with corresponding VA testing, which had to be performed no longer than 6 weeks prior or after the MRI. The study was approved by the institutional ethic committee (EA2/026/18) and conducted according to the good scientific practice guidelines of Charité Universitätsmedizin Berlin.

### MRI analysis

MRI data were heterogeneous with respect to field strength (1.5 and 3 T), MRI manufacturer (Siemens, Erlangen, Germany; General Electric Healthcare, Milwaukee, USA), MRI protocol, and contrast agent (gadoterate meglumine, gadobutrol). A prerequisite for inclusion were contrast enhanced T1-weighted, axial T2-weighted, and T2FLAIR-weighted series.

#### Tumor load measurements

**Fig. 1:**
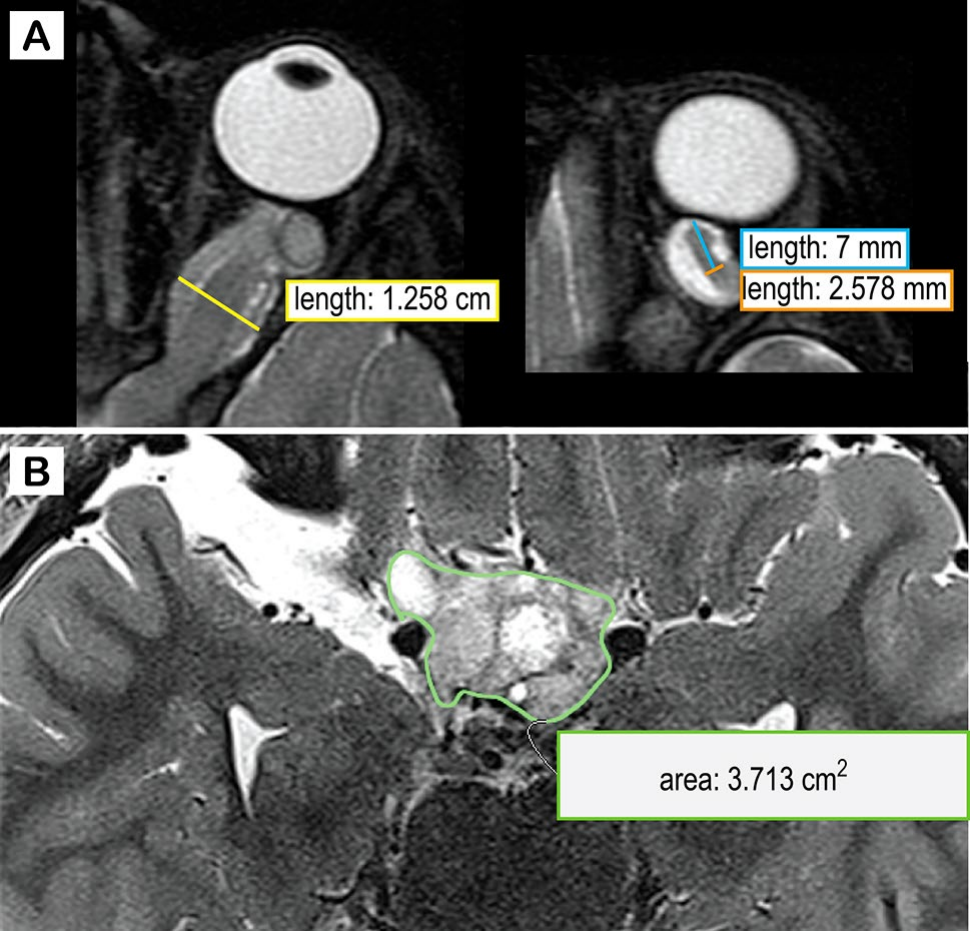
Assessment of tumor load. T2-weighted image of a 3 years old boy with neurofibromatosis type 1 and an optic pathway glioma. The optic nerve width was measured at its maximal width (yellow line in **A**) and 7 mm behind the globe (orange line in **A**). The intracranial tumor volume was determined by outlining the lesion on consecutive slice using the open source medical viewer Horos (Nimble Co LLC d/b/a Purview in Annapolis, MD USA). The inbuilt algorithm was used to calculate the overall tumor volume on the basis of the area and the slice thickness

MRI data were loaded into the open source medical viewer Horos (Nimble Co LLC d/b/a Purview in Annapolis, MD USA). The intraorbital optic nerve was measured (i) 7 mm behind the globe and (ii) at its maximal width (Fig. 1A). As described before [13], an optic nerve width > 3. 0 mm was defined as pathological. The intracranial tumor including the cisternal segment of the optic nerve, the chiasm, and the optic tracts was outlined on T2-weighted images using the pencil tool, and the volume was subsequently calculated by the inbuilt algorithm (Fig. 1B), hereafter termed intracranial tumor volume. Both was done by a medical student, trained by a neuroradiologist with long term experience in pediatric neuroradiology. All outlinings and optic nerve measurements were checked by the same neuroradiologist and adjusted if necessary. The medical student and the neuroradiologist were blinded for VA test results and prior or subsequent treatment.

#### Tumor enhancement score

The OPG was divided into 8 segments based on the modified Dodge classification [14]: the distal and proximal half of the intraorbital segment, the intracanalicular and the cisternal segment, the chiasm, the optic tract, the optic radiation, and the hypothalamus. If enhancement was present in one of the segments, 1 point was assigned. All structures could be counted twice, i.e. 1 point for the left, 1 for the right side, therefore adding up to a maximum of 16 points in extensively enhancing tumors. The assignment and calculation was carried out by the neuroradiologist, blinded for VA test results or treatment.

#### Tumor extension score

Anatomic tumor extension was assessed based on the modified Dodge classification [14]. In short, points were given concerning the following segments: intraorbital and cisternal (max. 4 points), chiasmatic (central or asymmetric left/right; max. 4), optic tracts (bilateral or asymmetric; max. 4), optic radiation (bilateral or asymmetric; max 3), and involvement of the hypothalamus (max. 1). Tumor involvement of multiple segments therefore results in a high tumor extension score. The assessment was done by the neuroradiologist, blinded for VA test results or treatment.

### Visual acuity testing

VA was evaluated with age-appropriate methods as part of the regular clinical visits, noted either as decimals or as Snellen fraction, and converted to the logarithmic minimal angle of resolution (logMAR) as described before [5]. Distance visual acuity (5 m) using optotypes (numbers) was measured if cooperation and mental capacity of the patient allowed it. In younger children, visual acuity was measured using LEA cards at 3 m (with matching if the child did not know the words for the symbols). In children unable to cooperate with these methods, the Cardiff Acuity Test (CAT, Cardiff University, Cardiff, UK) was used. In infants or if the child could not cooperate with the CAT, preferential looking was assessed using Teller Acuity Cards (Stereo Optical Company, Inc., Chicago, IL, USA).

### Statistical analysis

Statistical analysis was performed using R (Boston, MA, USA).

Median and interquartile ranges (IQR) of all first exams were calculated for NF1 + OPG and spOPG patients, respectively. Boxplots were generated for visualization. For bilateral measures (optic nerve width, logMAR, enhancement score), the mean of both sides and then the median and IQR for all exams were calculated.

The Spearman rank correlation between intracranial tumor volume as well as the maximal optic nerve width and enhancement scores were calculated taking repeated measurements on several occasions into account (rmcorr package [15]). Correlation coefficients are reported along with 95% confidence intervals (CI), separately for NF1 + OPG and SPOPG patients.

To examine which parameters influence VA, linear mixed models (lme4 package [16]) with random intercepts per patient and side affected were applied. Intracranial tumor volume, maximal optic nerve width, optic nerve width 7 mm behind the globe, enhancement score, tumor extension score, the patients’ age, time under observation, current chemotherapy, and sex were examined as potential explanatory variables. This was done for NF1 + OPG and spOPG patients separately. Regression coefficients are reported along with 95% confidence intervals (CI).

## Results

### Patients

Thirty-five OPG patients met the inclusion criteria, out of whom 22 (62.8%) had NF1. The proportion of females and the median age at first MRI were similar in NF1 + OPG and spOPG patients (36.4% and 38.5% males, respectively). Follow-up for NF1 + OPG was longer and their median age at first ophthalmological examination was higher (Table 1).

**Table 1:** Demographic data

Median (IQR)	NF1 + OPG (*n* = 22)	spOPG (*n* = 13)
Sex	8 (36%) males, 14 (64%) females	5 (39%) males, 8 (62%) females
Number of MRI studies per patient, median (IQR)	5 (2; 13)	6 (4; 10.5)
Median (IQR) of MRI observation period (years)	3.23 (1.40; 5.90)	1.85 (1.28; 5.31)
Median (IQR) age at first MRI (years)	4.74 (3.74; 5.67)	4.26 (0.99; 7.00)
Number of ophthalmological exams per case (IQR)	5 (2; 7)	4 (2; 9)
Median (IQR) age at first ophthalmological exam (years)	5.39 (3.35; 7.61)	1.81 (0.82; 6.88)
Number of patients having received chemotherapy	12 (55%)	9 (69%)
Median (IQR) age at start of chemotherapy (years)	7.25 (4.75; 9.21)	5.45 (3.23; 7.73)
Number of patients with tumor biopsy or partial resection	3 (14%)	6 (46%)
Median (IQR) age at surgery (years)	6.63 (4.41; 8.84)	4.94 (4.48; 5.84)

At some point during the observation period, a proportion of patients was treated with different chemotherapy agents. Fifteen children received vinblastine (10 of them with NF1 + OPGs), 12 vincristine/carboplatin (4 NF1 + OPGs), 2 vincristine/cisplatin/cyclophosphamide (one NF1 + OPG), 2 trametinib (one NF1 + OPG), 2 bevacizumab/irinotecan (spOPG), 2 thioguanine/procarbacin/CCNU/vincristine (spOPG), and 2 cisplatin/etoposide (no NF1 + OPG). Notably, some patients received different agents during the observation period. Biopsies or partial tumor resection was performed in 9 patients (3 NF1 + OPGs) (Table 1).

### MRI analysis

#### Tumor characteristics

Overall 298 MRI datasets were analyzed. Of 596 optic nerves, 32 could not be measured 7 mm behind the globe and 45 could not be measured at maximal width due to movement artefacts or sometimes artefacts caused by dental braces. The intracranial tumor volume was determined in all tumor positive MRI datasets. No metastatic disease was detected.

**Fig. 2:**
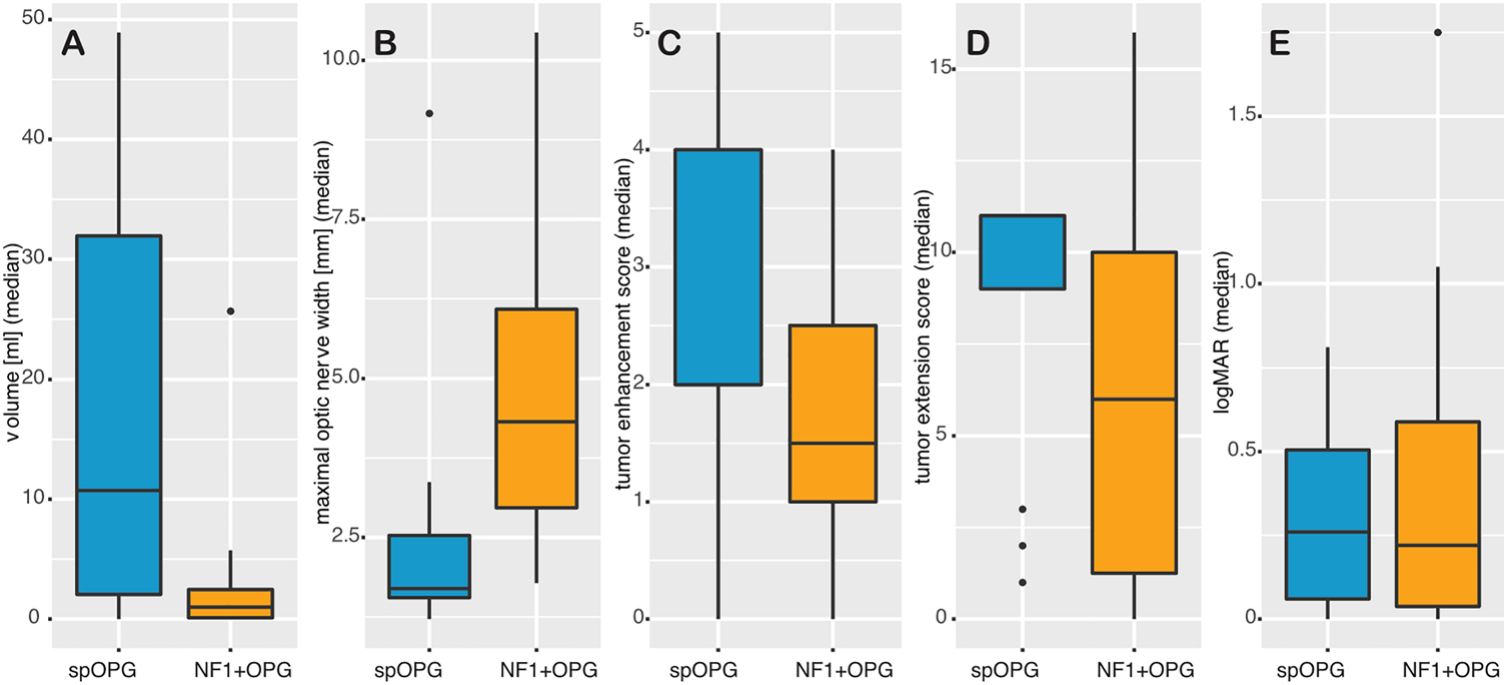
Intracranial tumor volume, maximal optic nerve, tumor enhancement score, tumor extension score, and visual acuity in logMAR in spOPG (blue) and NF1 + OPG (yellow) patients. The boxplots illustrate the distribution of the T2w tumor volume (**A**), the maximal optic nerve width (**B**), the tumor enhancement score (**C**), the tumor extension score (**D**), and the visual acuity measured in logMAR (**E**). The thick blue lines in the boxes are the median, the box height represents the 25th and 75th percentile, respectively. The whisker lengths are the min. and maximal values (25th and 75th percentile ± 1.5*interquartile range), dots potential outliers

106/182 MRI datasets (58%) of 16/22 NF1 + OPG patients (73%) showed intracranial tumor. Also, the majority of spOPG datasets were affected by intracranial tumor (114/116 (98%) in 12/13 (93%) patients). The median volume was 0.99 ml (IQR 0.1; 2.46 ml) in NF1 + OPG and 10.73 ml (2.05; 31.94 ml) in spOPG at study entry. spOPG patients rarely had an intraorbital tumor (pathological optic nerve width in only 13/232 (6%) optic nerves; median 1.7 mm, IQR 1.55; 2.53 mm), whereas the optic nerves were commonly enlarged in NF1 + OPG patients (262/364 (72%) optic nerves; median 4.32 mm, IQR 2.97; 6.08 mm). The data distribution is visualized by boxplots in Fig. 2A, B.

The median tumor enhancement score was 1.5 (IQR 1; 2.5) in NF1 + OPG and 2 (IQR 2; 4) in spOPG patients at first measurement (Fig. 2C).

The median tumor extension score was 6 (IQR 1.25; 10) in NF1 + OPG and 11 (IQR 9; 11) in spOPG patients, implying that spOPGs involve more segments of the optic pathway (Fig. 2D).

No relevant correlation was found between the enhancements scores and intracranial tumor volume in NF1 + OPGs (ρ 0.05, CI −0.11–0.2) or between the enhancements scores and maximal optic nerve width in spOPGs (ρ 0.05, CI −0.08–0.18). Enhancement scores correlated, however, moderately with the intracranial tumor volume in spOPGs (ρ 0.45, CI 0.28–0.59) and maximal optic nerve width in NF1 + OPG (ρ 0.49, CI 0.41–0.57).

#### Visual acuity and correlation with tumor characteristics

Median logMAR was 0.22 (0.04; 0.59) in NF1 + OPG and 0.26 (0.06; 0.5) in spOPG patients (Fig. 2E). No relevant correlation was found between VA and enhancement scores in both patient groups. Results are summarized in Table 2.

**Table 2:** Correlation coefficients and confidence intervals for ranks of tumor load and ranks of visual acuity with ranks of enhancement

Correlation coefficient ρ (CI)	NF1 + OPG (*n* = 22)	NF1-OPG (*n* = 13)
Intracranial tumor volume with enhancement scores	0.05 (−0.11–0.2)	0.49 (0.41–0.57)
Maximal optic nerve width with enhancement scores	0.45 (0.28–0.59)	0.05 (−0.08–0.18)
Visual acuity (logMAR) with enhancement scores	0.15 (0.05–0.25)	0.17 (0.04–0.30)

### Parameters associated with visual acuity (VA)

**Fig. 3:**
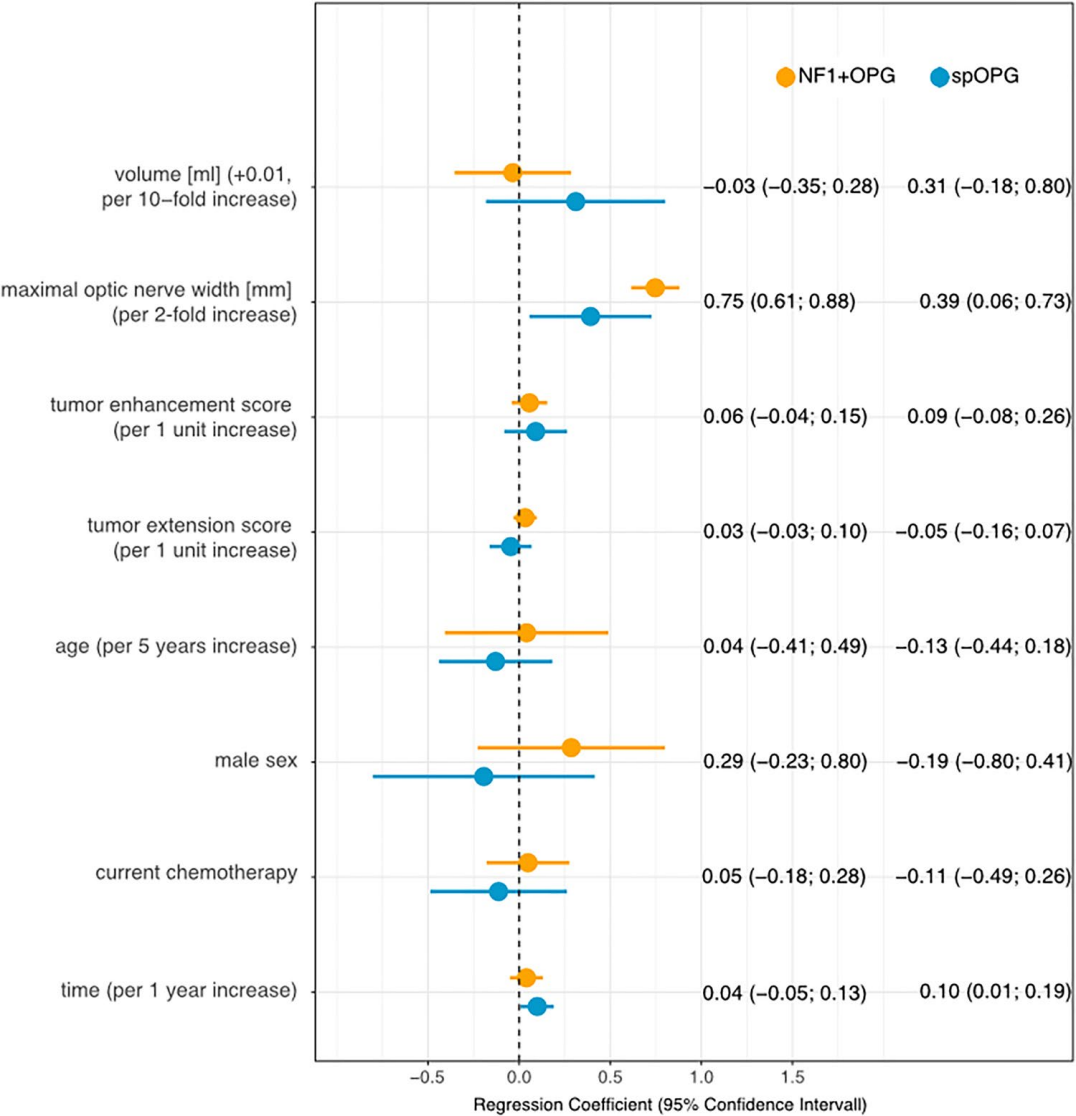
Parameters influencing visual acuity. Regression coefficients (95% confidence interval) for intracranial tumor volume, maximal optic nerve width, tumor enhancement score, tumor extension score, the patients’ age, sex, current treatment state, and overall observation time in patients with optic pathway gliomas and neurofibromatosis type 1 (NF1 + OPG, yellow) and for patients with sporadic optic pathway gliomas (spOPG, blue)

Investigating the potential effect of different variables on VA, a relevant association between the maximal optic nerve width with logMAR was shown in NF1 + OPG patients (regression coefficient 0.75; CI 0.61—0.88), while this was less pronounced in spOPGs (0.39; 0.05—0.73). This means that a twofold increase in nerve width was associated with a decrease of VA by 0.75 logMAR in NF1 + OPG, but only 0.39 in spOPG patients. In spOPGs, intracranial tumor volume correlated positively with VA (0.31; −0.19—0.81), which was not the case for NF1 + OPGs (0.03; −0.37—0.30). In both patient groups, the enhancement score was weakly associated with VA (spOPG: 0.09; −0.09—0.27; NF1 + OPG: 0.06; −0.04—0.15), i.e. an increase of the enhancement score by one point was associated with a decrease of VA by 0.09 logMAR in spOPGs and by 0.06 in NF1 + OPGs. The association of a longer observation period with worse VA for spOPGs (0.1; 0—0.20) allows suggesting that these patients might have a higher risk of visual acuity impairment over time, which was not the case for NF1 + OPG patients. For all other parameters investigated no correlation with VA was found. Results are summarized in Fig. 3. The model was repeated replacing maximal optic nerve width by the optic nerve width 7 mm behind the globe for both patient groups, but no significant change of the results was observed (Table A, suppl. material).

## Discussion

We investigated the diagnostic role of contrast enhancement in children with optic pathway gliomas with the aim to apply GBCAs more selectively during surveillance in these patients. First, a different tumor distribution pattern was found in NF1 + OPG and spOPG patients. While children with NF1 were commonly affected by intraorbital and smaller intracranial tumors, children with spNF1 had mostly larger intracranial tumors. Moreover, in spOPG patients more segments of the optic pathway were involved, i.e. the tumors were more extensive than in patients with NF1. In NF1 + OPG patients, a relevant association was only found between the maximal optic nerve width and contrast enhancement, while intracranial tumor load and VA did not correlate. In contrast, intracranial tumor volume and enhancement were associated in spOPG patients, while VA and the maximal optic nerve width did not show any relevant correlations. Since the most important parameter for treatment decisions in these children is their visual function, we aimed to elucidate predictive parameters for visual loss. Parameters affecting VA differed considerably depending on NF1 status. Although enhancement and maximal optic nerve width were correlated in NF1 + OPG patients, only the latter was found to affect the visual function. In spOPGs, the intracranial tumor load and the maximal optic nerve width were both associated with VA. In comparison, the tumor enhancement score was weakly correlated with VA in both patient groups, suggesting that its role may be secondary. Repetition of our analysis replacing the maximal optic nerve width by the nerve width 7 mm behind the globe showed similar results.

To date, only few studies have addressed the question if GBCAs are necessary when imaging patients with optic pathway tumors for diagnosis or treatment. Jittapiromsak et al. [6] examined 17 MRI datasets of 17 patients (12 NF1 + OPGs, 5 spOPGs). Differing to our results, they reported that progressive visual loss significantly correlated with diffuse contrast enhancement in contrast to a focal enhancement pattern that did not go along with visual loss and that probably corresponds to a low enhancement score in our study. Information regarding the NF1 status was not provided making a comparison with our results difficult. The influence of contrast enhancement, tumor volume, and/or visual loss on the decision to initiate treatment were investigated in three other, comparable studies, where tumor board and clinical notes were evaluated retrospectively [7, 8, 17]. An increase of tumor enhancement was never decisive in the single-center study by Maloney et al. [7], whereas it was found to be a co-factor in 20% of patients in the multi-center study by Fisher et al. [17]. The recent study by Malbari et al. confirms these findings, i.e. that contrast enhancement almost never led to a change of management [8]. Corroborating our results, the authors of these studies suggest that the overall tumor load rather than the contrast enhancement matters regarding treatment decisions, in their case as basis for decision-making, in our case as a predictor for visual function. The relevance of our study lies in the more quantitative approach, e.g. VA testing results, dedicated volume measurements, enhancement scores, and tumor extension scores that altogether facilitates a transfer between centers. One might argue that tumors are easier to delineate and measure on GBCA-enhanced MRI series, but this could not be confirmed in a recent study by Maloney et al. assessing intra- and inter-rater agreement of tumor measurements [18]. Here, an added value of GBCA-enhanced series was found in only a minority of cases (17.3%), but mostly due to thinner slices that resulted in a higher confidence. This could be easily accommodated by adjusting the imaging protocol and would not justify regular GBCA administration. The same was found by Malbari et al. [8]. The general tumor distribution in our patients corresponds to that described in the literature [14, 18, 19]; NF1 + OPGs are mostly located in the orbital segments of the optic nerve and may extent into the cisternal segments, the optic chiasma, and tracts, while spOPGs arise most often in the chiasma and hypothalamus.

In contrast to Taylor et al. and Fisher et al. who retrospectively evaluated tumor board notes, we preferred visual function as outcome parameter for our analysis, because VA expressed in logMAR is a validated and reproducible functional assessment score [5]. Additionally, treatment is often initiated based on a combination of clinical symptoms and imaging findings [17], which complicates the retrospective determination of single or dominant factors. Moreover, parameters prompting the initiation of treatment might differ between centers as shown by Fisher et al. [17], which makes the translation of findings challenging. As the preservation of the visual function is the central goal in the decision-making process, we regarded VA as the most relevant parameter [12, 20, 22].

Response criteria for treatment are well established for high-grade and low-grade tumors in adults and children [12, 23, 24]. The recent criteria by the RAPNO working group emphasizes that especially in NF1 + OPG patients visual impairment rather than tumor growth is the most common symptom leading to therapy [12]. Contrast administration is not mentioned in this context, but is still recommended as a standard for response assessment in optic pathway gliomas, but without specifying details. It is, however, well recognized that pediatric low-grade glioma show variable and fluctuating enhancement without the significance of this being ultimately understood [25, 26]. Moreover, decrease of contrast enhancement during chemotherapy, treatment with bevacizumab or mitogen-activated protein kinase kinase (MEK) inhibitors is well known, but is not necessarily evidence of response [27], which might be the reason why it is no longer included as response criteria in clinical trials for pediatric glioma [20, 28]. Regular GBCA administration might therefore be debatable in optic pathway gliomas. This does, however, not apply to NF1 patients suspected of having gliomas outside the optic pathway, but this was not subject of our study.

The limitations of this study include its retrospective nature and the fact that MRI and ophthalmological data were collected over a time period of 16 years. During this time, MRI systems and data quality have changed considerably. We have included MRI data from both 1.5 and 3 Tesla systems with consequently heterogeneous scan parameters. Further on, we did not consider differences of GBCAs (gadobutrol and gadoterate meglumine in our data) and we were not able to allow for differences in delay between administration and image acquisition. All of this might have some relevance for the optic nerve width, the intracranial tumor volume, and the enhancement score. Since OPGs are often irregular and insufficiently detected by linear measurements, we measured intracranial tumor load using a volumetric approach instead of the recommended linear measurements in two or three planes [12]. Although we regard this as a strength of our study, volumetric assessment is currently not viable in a clinical context, as dedicated software is not widely available. VA assessment in children should be performed with age-appropriate methods and may be flawed by lack of cooperation. In children with NF1, neurocognitive deficits are more prevalent, and care should be taken to use more appropriate methods for an individual patient. Due to the aforementioned retrospective nature of our study, VA may have been assessed in different manners and by different examiners or, as the child gets older, with different tests. Still, because this study was conducted at a single center, the methods were rather consistent. Additional parameters regarding visual function such as visual fields may also provide valuable information, as may morphological measurements, e.g. retinal nerve fiber layer thickness as measured by optical coherence tomography (OCT). First studies have shown that especially OCT has great potential to monitor disease in NF1-related OPGs [29, 30], but OCT results were only available for later time points in our cohort, why they were not included in our analysis. Correlation of OCT with imaging findings would be highly relevant and this should be done in future studies. Moreover, patients were in different treatment states during the observation period; some patients were treatment-naïve, some MRI data were collected during, others after treatment. As chemotherapy, treatment with bevacizumab or MEK inhibitors can lead to decreased enhancement [27, 28], this is a potential confounder. We therefore assessed the effect of the enhancement score on VA, adjusted by the different parameters, i.e. age, sex, observation time, current treatment, tumor volume, and maximal optic nerve width, and found that only the maximal optic nerve width changed the effect relevantly, regardless of the NF1 status, emphasizing the importance of this parameter over that of enhancement for VA. We neglected surgical interventions in our analysis, since they were done in only a minority of patients and consisted mainly of biopsies or partial resections.

In conclusion, our results indicate that GBCA administration for surveillance MRI in OPG patients is not generally necessary, given that VA is the pivotal outcome criteria. Tumor load measurements on T2-weighted images, in particular of the optic nerve width, may be sufficient in both NF1 + OPGs and spOPGs. Although a minimal association between enhancement and VA was found, we believe that this does not justify regular GBCA administrations in these patients.

## Supplementary Information

Below is the link to the electronic supplementary material.

Supplementary file1 (DOCX 14 kb)

## Data Availability

The datasets generated during and/or analysed during the current study are available from the corresponding author on reasonable request.
