# Supplementary material for: Tumor load rather than contrast enhancement is associated with the visual function of children and adolescents with optic pathway glioma – a retrospective Magnetic Resonance Imaging study
Source: J Neurooncol. 2022 Jan 7;156(3):589–97. doi: 10.1007/s11060-021-03941-1 (PMC8860805; doi:10.1007/s11060-021-03941-1)
Supplement: Supplementary file 1 — Supplementary file1 (DOCX 14 kb) [file 11060_2021_3941_MOESM1_ESM.docx]

| Tab. A: Regression coefficients (95% confidence intervals) assessing the effect of different parameters on the visual acuity with random intercepts per patient and side affected. | | |
| --- | --- | --- |
| Regression coefficient (CI) | NF1+OPG (n = 22) | NF1-OPG (n = 13) |
| intracranial tumor volume in ml (+0.01; per 10-fold increase) | -0.10 (-0.42 - 0.23) | 0.24 (-0.26 - 0.74) |
| optic nerve width 7 mm behind the globe in mm (per 2-fold increase) | 0.78 (0.64 - 0.91) | 0.26 (-0.22 - 0.74) |
| tumor enhancement score (per 1 unit increase) | 0.06 (-0.03 - 0.16) | 0.31 (0.02-0.32) |
| tumor extension score (per 1 unit increase) | 0.07 (0.01 - 0.13) | -0.08 (-0.19 - 0.04) |
| age (per 5 years increase) | 0.25 (-0.21 - 0.71) | -0.12 (-0.45 - 0.20) |
| male sex | 0.22 (-0.32 - 0.76) | 0.00 (-0.63 - 0.63) |
| current chemotherapy | 0.04 (-0.18 - 0.26) | -0.12 (-0.50 - 0.26) |
| time (per 1 year increase) | 0.00 (-0.10 - 0.09) | 0.10 (0.00 - 0.19) |
